# The disease burden of ocular toxoplasmosis in Denmark in 2019: Estimates based on laboratory testing of ocular samples and on publicly available register data

**DOI:** 10.1016/j.parepi.2021.e00229

**Published:** 2021-10-12

**Authors:** Jonathan Marstrand, Jørgen Anders Lindholm Kurtzhals, Helle Josefine Fuchs, Henrik Vedel Nielsen, Pikka Jokelainen

**Affiliations:** a Centre for Medical Parasitology, Department of Immunology and Microbiology, University of Copenhagen, Copenhagen, Denmark; b Department of Clinical Microbiology, Copenhagen University Hospital (Rigshospitalet), Copenhagen, Denmark; c Department of Ophthalmology, Copenhagen University Hospital, Copenhagen, Denmark; d Laboratory of Parasitology, Department of Bacteria, Parasites & Fungi, Infectious Disease Preparedness, Statens Serum Institut, Copenhagen, Denmark

**Keywords:** DALY, Disease burden, Ocular toxoplasmosis, *Toxoplasma gondii*, Toxoplasmosis

## Abstract

**Background:**

*Toxoplasma gondii* is an important zoonotic protozoan parasite with worldwide distribution. Information on the contribution of ocular toxoplasmosis to the disease burden caused by this parasite is limited or lacking from many countries.

**Methods:**

We estimated the minimum occurrence of ocular toxoplasmosis in Denmark using results from direct detection of *T. gondii* DNA with qPCR and determination of the Goldmann-Witmer coefficient on ocular samples submitted by ophthalmological clinics and departments to the national reference laboratory in 2003–2019. In addition, we inferred incidence estimates using retrospective data that are publicly available in the National Patient Register, and we used unstructured expert elicitation as the basis for sensitivity analyses. We estimated the disease burden of ocular toxoplasmosis in 2019 in disability-adjusted life years (DALYs).

**Findings:**

Ocular samples from 263 individuals (median age 57 years, range 2–88) had been tested with at least one of the methods during 2003–2019, and 42 (16%) tested positive (median age 65 years, range 14–85). In 2019, five (16%) of 31 tested individuals were positive, giving a minimum annual incidence estimate of 0.09 per 100.000 population. From this, we calculated a disease burden of at least 4 DALYs (95% confidence interval, 3–5). The age range suggested that this figure represented postnatally acquired ocular toxoplasmosis. The disease burden of ocular toxoplasmosis due to congenital toxoplasmosis has been previously estimated to be at least 12 DALYs, resulting in an estimated minimum total disease burden due to ocular toxoplasmosis of 16 DALYs. In 2005–2018, the mean annual number of diagnoses of ocular toxoplasmosis reported to the National Patient Register was 186, and the corresponding disease burden estimate was 134 DALYs (95% confidence interval, 113–158). Sensitivity analyses focusing on incidence and severity resulted in disease burden estimates in the range of 9–523 DALYs.

**Interpretation:**

Because most diagnoses of ocular toxoplasmosis are based on clinical observations, ophthalmoscopy, and serology without confirmatory testing, the disease burden caused by ocular toxoplasmosis is likely substantially higher than our minimum estimates. Our results indicate that ocular toxoplasmosis contributes to the disease burden caused by *T. gondii* in Denmark, but uncertainty about the incidence and severity precludes reliable estimation of its importance.

## 1 Introduction

The zoonotic protozoan *Toxoplasma gondii* can infect humans and other warm-blooded animals, congenitally or later in life (Dubey, 2010; Moncada and Montoya, 2012; Montoya and Liesenfeld, 2004; Robert-Gangneux and Dardé, 2012). In Denmark, immunoglobulin G antibodies against *T. gondii* have been reported in 27% of pregnant women (Lebech et al., 1993) and in a substantial proportion of animal hosts (Henriksen et al., 1994; Kofoed et al., 2017; Laforet et al., 2019; Nielsen and Wegener, 1997; Nielsen et al., 2019; Olsen et al., 2019), together indicating that the parasite is common and endemic in the country.

In most immunocompetent individuals, *T. gondii* infection is subclinical or causes unspecific clinical signs and symptoms such as fever, fatigue, and lymphadenopathy (Ben-Harari and Connolly, 2019; Yates et al., 2019). However, the parasite can also cause severe clinical manifestations, leading to a substantial disease burden (Ben-Harari and Connolly, 2019; Garweg, 2016; Kortbeek et al., 2009; Nissen et al., 2017). Most of the disease burden estimates have focused on congenital toxoplasmosis, including ocular toxoplasmosis as one of its typical manifestations (Lebech et al., 1993; Kortbeek et al., 2009; Nissen et al., 2017; Abu et al., 2016; Bustillo et al., 2015; de-la-Torre et al., 2009; Gilbert et al., 2001; Glasner et al., 1992; Havelaar et al., 2012; Havelaar et al., 2007; Jones and Holland, 2010; Pires et al., 2020; Röser et al., 2010; Schmidt et al., 2006; Takeda et al., 2017). Ocular toxoplasmosis can also be acquired later in life, but the disease burden caused by acquired ocular toxoplasmosis has only been estimated in few studies, none of which have been performed in the Nordic countries (Dubey, 2010; Moncada and Montoya, 2012; Montoya and Liesenfeld, 2004; Robert-Gangneux and Dardé, 2012; Dubey et al., 2012; Peyron et al., 2017).

There are very few publications about ocular toxoplasmosis in Denmark, most of which focused on congenital toxoplasmosis and a single study investigated genetic variation of *T. gondii* in clinical samples (Jokelainen et al., 2018). Two of these papers estimated the disease burden of congenital toxoplasmosis at 123 and 165 disability-adjusted life years (DALYs), including 12 and 30 DALYs due to ocular toxoplasmosis, respectively (Nissen et al., 2017; Pires et al., 2020). No estimates of the disease burden caused by postnatally acquired ocular toxoplasmosis nor the total disease burden of ocular toxoplasmosis have been made in Denmark. Such estimates are necessary to estimate the overall disease burden caused by the parasite, and thus to better inform public health decisions. This study aimed to estimate the total disease burden of ocular toxoplasmosis in Denmark.

## 2 Materials and methods

### 2.1 Setting

Denmark is a Nordic country with a population of 5.8 million people (Danmarks Statistik, 2020a) and a life expectancy of 81.3 years (Danmarks Statistik, 2020b). Toxoplasmosis is not a notifiable disease in Denmark. The Danish National Patient Register contains information on all diagnoses registered for patients in contact with a hospital in the country (Sundhedsdatastyrelsen, 2020).

The national guidelines recommend that ocular toxoplasmosis is managed by specialists (Sundhed, 2019). Most cases are diagnosed based on clinical symptoms, ophthalmoscopy, and serology. Confirmatory testing for ocular toxoplasmosis is available at the national reference laboratory for parasites at Statens Serum Institut, Copenhagen, Denmark. The laboratory offers real-time PCR testing for *T. gondii* DNA (Jokelainen et al., 2018; Homan et al., 2000). The service is used by four of five regions in Denmark, whereas the last region performs the test locally. Determination of the Goldmann-Witmer coefficient (Prüss-Üstün et al., 2003; De Groot-Mijnes et al., 2006) for detecting a local immunological response against the parasite is only available via the national reference laboratory and has been available since 2015.

### 2.2 Estimating the disease burden of ocular toxoplasmosis in Denmark

#### 2.2.1 Incidence of ocular toxoplasmosis

To estimate the incidence of ocular toxoplasmosis in Denmark, we used a retrospective anonymized extract of specific laboratory test results from the national reference laboratory and retrospective publicly available data from the National Patient Register.

#### 2.2.2 Test results from the national reference laboratory

The extract included results (negative or positive) of qPCR and determination of the Goldmann-Witmer coefficient performed on ocular samples that had been submitted from ophthalmological clinics and departments in 2003–2019, together with the year when each sample was received in the laboratory, patient gender and age at sampling, type of sample material and for some samples brief accompanying clinical notes, from which we only extracted if ‘retinitis’ or ‘uveitis’ was mentioned. We considered a positive result in at least one of the two tests as confirmatory for ocular toxoplasmosis. For those with several test results during the 17-year period, we included one. If all test results were negative, the latest result was included; if one or several test results were positive, we included the first positive result.

#### 2.2.3 Data from the National Patient Register

The total annual number of registered diagnoses of ocular toxoplasmosis, International Statistical Classification of Diseases and Related Health Problems (ICD-10) codes DH320D and DB580, was extracted from the National Patient Register for years 2005–2018. This fourteen-year period includes the most recent data available within the period covered by the laboratory result extract.

#### 2.2.4 Estimates of the disease burden of ocular toxoplasmosis

We estimated the disease burden of ocular toxoplasmosis for the latest available year in which 1) the number of laboratory tests was above the mean number of annual tests performed in 2003–2019, and 2) the proportion of positive test results for the year did not differ significantly from the proportion in 2003–2019.

We estimated the disease burden in DALYs (Prüss-Üstün et al., 2003; Devleesschauwer et al., 2014; WHO, 2020). The minimum laboratory-based estimate of the incidence was based on the number of individuals with a positive test result during the year. The register-based estimate of the incidence was based on the mean annual number of registered diagnoses of ocular toxoplasmosis in all the data extracted from the National Patient Register. We assumed that individuals with ocular toxoplasmosis had moderate chorioretinitis with a disability weight of 0.033 (Foodborne Disease Burden Epidemiology Reference Group (FERG), 2015) and did not have reduced life span. To calculate the expected number of years lived with disability, we subtracted the mean age at sampling of positive test results from the life expectancy (Danmarks Statistik, 2020b). For this, we used aggregated data from the national reference laboratory from all the years (2003–2019) because the number of individuals with a positive test result per year was low. Furthermore, we evaluated the age distribution of the individuals tested to see if the infections were likely to be acquired after birth. To estimate the total disease burden of ocular toxoplasmosis, we merged our estimate with a previous estimate for ocular toxoplasmosis caused by congenital toxoplasmosis (Nissen et al., 2017). We performed sensitivity analyses based on an unstructured expert elicitation, making use of the local knowledge of the authors. The main aspects of the expert elicitation were the proportion of cases of ocular toxoplasmosis that would be diagnosed at the national reference laboratory and the severity of such cases.

#### 2.2.5 Statistical analyses

Descriptive statistics (mean, median, percentage) were calculated in Microsoft Excel. We calculated 95% confidence intervals (95% CI, Mid-*P* exact) for proportions and evaluated differences between proportions (two-by-two tables, 2-tailed *P*-value, Mid-*P* exact) using the open-source software OpenEpi (Sullivan et al., 2020). We considered *P* < 0.05 to indicate statistical significance. We evaluated the agreement between the tests by calculating percent agreement and Kappa using Stata 13.1 (Stata Corporation, TX, USA). We calculated the 95% confidence interval for the disease burden estimates using RStudio (version 1.2.5033).

#### 2.2.6 Ethical considerations

The test result extract was anonymized, and the study included no contact with the individuals who had been tested. We used publicly available aggregated data of annual total number of diagnoses of ocular toxoplasmosis from the National Patient Register (eSundhed, 2020). We handled, and present, all data as aggregated summaries, in a way that the individuals cannot be identified. The data are presented so that they cannot be cross tabulated to combine information by the variables. No details (n positive, 95% CI of the proportion) are shown for categories with fewer than 20 individuals to ensure anonymity. The publication only contains aggregated results and no personal data and is therefore not covered by the European General Data Protection Regulation.

## 3 Results

**Fig. 1:**
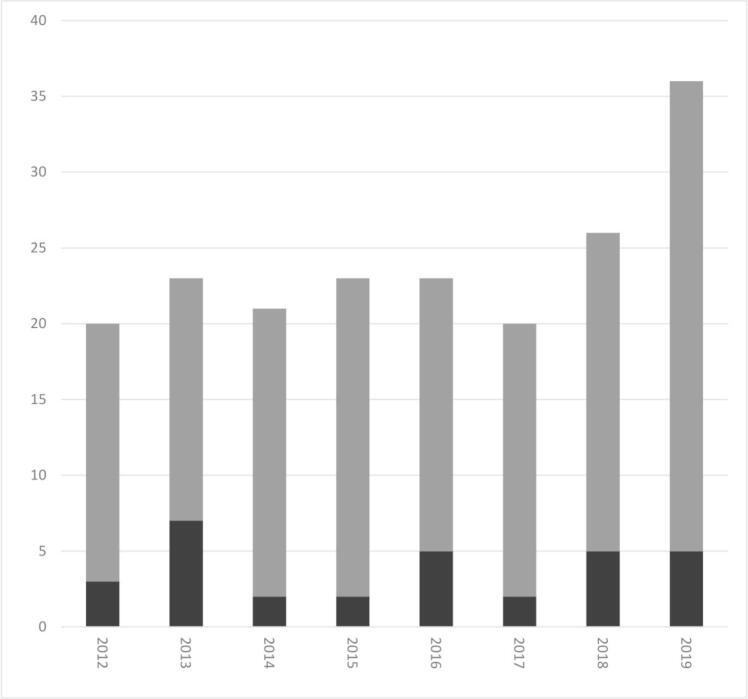
Number of positive test results (black) and negative test results (gray) from qPCR and/or calculation of the Goldmann-Witmer coefficient for *Toxoplasma gondii* on ocular samples at the national reference laboratory for parasites, Denmark, 2012–2019, by year.

The extract from the national reference laboratory consisted of 299 test results. Thirty-six (12%) results were excluded due to repeated testing. The final dataset comprised test results for 263 individuals. Most of the included samples were received in 2012–2019 (Fig. 1); the number of samples per year in 2003–2011 was below 20. Of the 263 individuals, 121 (46%) were females and 142 (54%) were males. The age range was 2–88 years (mean, 55 years; median, 57 years). All samples for which the Goldmann-Witmer coefficient was determined were also tested by qPCR.

A total of 42 (16%) of the individuals tested positive in at least one of the two tests. Sixteen percent (41/263) had a positive qPCR result and 7% (4/54) had positive Goldmann-Witmer coefficient result. Of the 54 individuals who had been tested by both methods, the results agreed for 49. Forty-six were negative with both, three were positive with both, four had a positive qPCR result, and one had positive Goldmann-Witmer coefficient result. The percent agreement was 91%, and Kappa was 0.5.

The age at positive test ranged from 14 to 85 years (mean, 60 years; median, 65 years). The proportion of positive results varied from 0% to 33% by age group (Table 1) and did not show difference by gender (Table 1). Most of the samples were taken from corpus vitreum, and ‘uveitis’ was often mentioned in the clinical notes (Table 1).

**Table 1:** Number and proportion of included individuals and their samples that were positive with qPCR and/or calculation of the Goldmann-Witmer coefficient performed for detecting *Toxoplasma gondii* DNA or immunological response against *T. gondii* on ocular samples at the national reference laboratory for parasites, Denmark, 2003–2019.

	N total	n positive	% positive	95% CI
Age group				
≤9 years	<20^a^		0	
10–19 years	<20		33	
20–29 years	26	3	12	3–28
30–39 years	<20		11	
40–49 years	44	4	9	3–20
50–59 years	43	3	7	2–18
60–69 years	52	13	25	15–38
70–79 years	52	11	21	12–34
≥80 years	<20		21	

Gender				
Female	121	18	15	9–22
Male	142	24	17	11–24

Sample material				
Anterior chamber	57	7	12	6–23
Corpus vitreum	178	25	14	10–20
Other or unspecified	28	10	36	20–54

Clinical notes				
Retinitis^b^	<20		40	
Uveitis^b^	95	11	12	6–19
Other or none	159	27	17	12–23

Total	263	42	16	12–21

CI, confidence interval.

a No details (n, 95% CI) are shown for categories with fewer than 20 individuals, to ensure anonymity.

b Both mentioned for one sample.

The number of individuals with a positive test result varied from zero to seven (mean, 2.5; median, 2) per year, and the proportion of positive tests varied from 0% to 50% (mean, 19%; median, 16%). In 2019, five (16%) of the 31 results were positive. Thus, the criteria for making the disease burden estimate for the year 2019 were met: the number of tests performed in 2019 was higher than the annual mean of 15 during 2003–2019 and the proportion of positive test results in 2019 did not differ significantly from the proportion in 2003–2019 (16% and 16%, respectively, *P* = 0.9).

The five individuals with a positive test result in 2019 was translated to a minimum incidence estimate in 2019 of 0.09 per 100.000 population per year. The mean age at first positive test during 2003–2019 was 60 years, and the number of years living with the disease was thus expected to be 21.3. We estimated the minimum disease burden to be four DALYs (95% CI, 3–5).

The individuals who tested positive were aged 14 years and older, and we thus considered them to mainly represent acquired ocular toxoplasmosis. Together with a previous estimate of 12 DALYs for ocular toxoplasmosis associated with ocular toxiplasmosis (Nissen et al., 2017), this gave a minimum total disease burden caused by ocular toxoplasmosis in Denmark of 16 DALYs.

The National Patient Register had records of a total of 2610 diagnoses of ocular toxoplasmosis during the time period (eSundhed, 2020). Based on this, the mean annual number of registered diagnoses of ocular toxoplasmosis was 186. This count gave an incidence estimate of 3.2 per 100,000 population per year (Danmarks Statistik, 2020a). A disease burden estimate based on this figure as incidence estimate, while not changing the other parameters, was 134 DALYs (95% CI, 113–158).

The main conclusion of the expert elicitation was a high level of uncertainty regarding the incidence. This uncertainty was considered mainly to be due to the large proportion of cases diagnosed in specialist practice based on clinical findings by ophthalmoscopy supported by detection of anti-*T. gondii* antibodies in peripheral blood. Our expert elicitation indicated that perhaps <10% of all cases of ocular toxoplasmosis in Denmark would be included in the laboratory-based minimum estimate of the incidence for 2019. This was in line with the available data: the mean annual number of positive test results was 1.3% of the mean annual number of registered diagnoses (2.5 and 186, respectively). Furthermore, the cases diagnosed at the national reference laboratory were considered likely to be more severe, cryptic, or complicated than cases identified by other approaches. Finally, for estimates based on register data it was noted that ocular toxoplasmosis may be reported several times for the same individual in the register. Conversely, ocular toxoplasmosis might go unreported when congenital toxoplasmosis is reported to the register. Underreporting was considered possible. A previous study on congenital toxoplasmosis in Denmark concluded that for each reported case of congenital toxoplasmosis, 2–7 other cases of congenital toxoplasmosis could be expected to have occurred (Nissen et al., 2017). We did not have data indicating whether these aspects might have led to overestimation or underestimation of the incidence and burden.

Based on the expert elicitation we conducted sensitivity analyses using 50, 150, and 300 as the possible numbers of cases of ocular toxoplasmosis in 2019. These yielded disease burden estimates of 36, 108, and 216 DALYs, respectively. We also did a disability weight -adjustment considering that reported cases might be more severe. For this we used a disability weight of 0.08 (Kortbeek et al., 2009) resulting in a minimum disease burden of nine DALYs, a minimum total disease burden caused by ocular toxoplasmosis of 21 DALYs, and a disease burden based on registered cases of 325 DALYs. Disability weight -adjusted estimates for 50, 150, and 300 annual cases yielded disease burden estimates of 87, 261, and 523 DALYs, respectively.

## 4 Discussion

This is the first study estimating the disease burden of ocular toxoplasmosis and summarizing the available data on the disease in Denmark. In our most conservative estimate, acquired ocular toxoplasmosis caused an annual disease burden of at least four DALYs. Adding this to the burden of ocular toxoplasmosis related to congenital toxoplasmosis resulted in a minimum total disease burden of 16 DALYs. The estimate based on register data was 134 DALYs. Sensitivity analyses addressed a potential underestimation of the incidence and risk of referral bias towards reporting of severe cases and suggested that the disease burden of ocular toxoplasmosis could be substantially higher than the minimum estimates.

The main gap in the data that prevented an accurate estimation of the disease burden was the lack of a good estimate of the incidence of acquired ocular toxoplasmosis. As ocular toxoplasmosis is not a notifiable disease and there is no active surveillance, the incidence estimates remain uncertain. Prospective studies, using standard definitions and including both hospital departments and private practice are needed in order to obtain reliable data on the incidence of and disease burden caused by ocular toxoplasmosis. Another challenge was lack of sufficient data for a specific year, which we addressed by using data from longer time periods.

For our minimum estimate of the incidence, we used a laboratory-based approach with data from the national reference laboratory, which receives samples from four out of five regions i.e. did not cover the whole country. Our expert elicitation indicated that a considerable number of cases were likely to have been missed due to variation in diagnostic approaches. As ocular toxoplasmosis covers several clinical manifestations, some of which are unspecific, and the diagnosis can be reached by different approaches (Vasconcelos-Santos et al., 2011), the minimum estimate of the incidence is clearly an underestimate of the actual incidence.

To capture cases diagnosed by the different approaches, we used a register-based approach and employed available data from the National Patient Register. However, the register only registers data from hospitals and not private practice, and underreporting was considered possible, e.g., when ocular toxoplasmosis was part of a combination of manifestations. We did not have data to estimate how much these aspects could bias the results towards underestimation. Conversely, the same person could have been registered in the register several times, as the data cover all ambulatory visits with the specific diagnosis registered. Due to lack of data on the recurrence rate in the Danish setting we could not estimate how the possible repeated entries could bias the results towards overestimation. A recent study from France reported 0.11 recurrences after severe ocular toxoplasmosis episode per patient per year (Matet et al., 2019), which was similar to the rate reported from Australia (Yates et al., 2019) but lower than what was observed in Colombia (Velasco-Velásquez et al., 2021).

For our laboratory-based approach, we combined the results of two laboratory tests to increase sensitivity. However, in our sample, determination of the Goldmann-Witmer coefficient added only a single positive result that was not captured by qPCR. The annual number of individuals tested increased during the years, which could reflect changes in diagnostic approaches used in the clinical setting. While the reasons for submitting the samples for testing remain unknown, referral bias was expected to be substantial and could affect the results. There was no indication of substantially different magnitude of bias in 2019 when compared with other years: the proportion of positive results in 2019 was in line with the proportion during the whole period 2003–2019.

The mean age at a positive laboratory result was 60 years, and the age range at positive laboratory results indicated that individuals who tested positive likely had acquired ocular toxoplasmosis, rather than ocular toxoplasmosis related to congenital toxoplasmosis. However, we had no data to corroborate this assumption. Other studies have reported a median age of 35.5 years in individuals diagnosed with ocular toxoplasmosis (Yates et al., 2019) and a mean age of 21.7 years in individuals diagnosed with acquired ocular toxoplasmosis (Garweg, 2016; Delair et al., 2008). Whether the use of intraocular diagnostic sampling or diagnostic approach to ocular toxoplasmosis varies by age group is unknown but it might explain the difference. Regardless, our results suggest that ocular toxoplasmosis may merit more attention in older age groups.

The disability weight used in the calculations also affects the disease burden estimates, as seen from the results of our sensitivity analyses. Local data on disease severity, in particular of acquired ocular toxoplasmosis, was unavailable. Milder cases were probably underrepresented in the laboratory-based estimate, and the diversity of *T. gondii* strains detected in clinical samples in Denmark appears to be high (Jokelainen et al., 2018), which may support the use of a higher disability weight in the sensitivity analyses. Strains that are atypical in Europe have been associated with more severe ocular toxoplasmosis (De-La-Torre et al., 2013; Gilbert et al., 2008). Furthermore, if testing using the two methods was used more commonly for older individuals, as the total numbers tested per age group suggest (Table 1), the laboratory-based estimate could be an underestimation of the disease burden. Individuals affected at a younger age might be underrepresented in the laboratory-based estimate, and they live with the disability for longer.

The contribution of ocular toxoplasmosis to the total burden of eye diseases remains to be estimated. The global burden of eye disease has been estimated to be altogether 61.4 million DALYs and to be unevenly distributed across the world (Ono et al., 2010). Further aspects, such as psychosocial aspects related to ocular toxoplasmosis and quality of life after the diagnosis should also be considered (Canamary Jr et al., 2020).

Our work identified data gaps, and thus it can be concluded that ocular toxoplasmosis merits more attention in Denmark. Studies that would help the most in estimating the disease burden caused by ocular toxoplasmosis more accurately include studies yielding 1) estimates of the incidence, 2) data on age distribution at diagnosis, and 3) applicable estimates of severity. A prospective cohort study would be optimal to fill the main gaps.

## 5 Conclusions

Our results allow us to conclude that ocular toxoplasmosis contributes to the overall disease burden caused by *T. gondii* in Denmark. However, an accurate estimate of the disease burden cannot be calculated in the absence of reliable data on incidence. More studies on ocular toxoplasmosis in the country, and the region, are needed.

## Funding

PJ and HVN are part of the TOXOSOURCES consortium, supported by funding from the 10.13039/501100000780European Union's 10.13039/100010661Horizon 2020 Research and Innovation programme under Grant Agreement No 773830: One Health European Joint Programme.

## Declaration of Competing Interest

PJ is Editor of Parasite Epidemiology and Control. The authors declare that they have no other potential conflict of interest.
